# A Bayesian Approach to Sport Injuries Likelihood: Does Player’s Self-Efficacy and Environmental Factors Plays the Main Role?

**DOI:** 10.3389/fpsyg.2018.01174

**Published:** 2018-07-06

**Authors:** Aurelio Olmedilla, Víctor J. Rubio, Pilar Fuster-Parra, Constanza Pujals, Alexandre García-Mas

**Affiliations:** ^1^Department of Personality, Assessment and Psychological Intervention, University of Murcia, Murcia, Spain; ^2^Department of Biological and Health Psychology, School of Psychology, University Autonoma of Madrid, Madrid, Spain; ^3^Department of Mathematics and Computer Science, University of the Balearic Islands, Palma de Mallorca, Spain; ^4^Department of Psychology, Faculdade Ingá/UNINGA, Maringá, Brazil; ^5^Department of Basic Psychology, University of the Balearic Islands, Palma de Mallorca, Spain

**Keywords:** injuries likelihood, psychological triggers and consequences, self-efficacy, sport injuries, Bayesian networks

## Abstract

The psychological factors of sports injuries constitute a growing field of study, even from the point of view of the prediction of their occurrence. Most of them, however, do not take into account the likelihood of the injuries’ occurrence and the weight and role of the psychological variables on it. We conducted a study building up a Bayesian Network on a big sample of athletes, trying to assess these probabilistic links among several relevant psychological variables and the injuries’ occurrence. The sample was constituted by 297 athletes (239 males, 58 females) from a wide range of sports: track and field; judo; fencing; karate; boxing; swimming; kayaking; artistic rollerskating, and team sports as football, basketball, and handball (Mean age: 25.10 ±-3.87; range: 21–38 years). Several psychological variables, such as anxiety, social support, and self-efficacy were studied. Also, we recorded the history of injuries as well the body mass index and personal epidemiological data. The overall picture of the generated graph and Bayesian Network and its analysis – including the use of hypothetical data by means of several instantiations – includes the nuclear role of the Self-Efficacy regarding the injuries’ occurrence likelihood; the decreasing impact of the competitive anxiety previous to the injury; the probabilistic independence of the players’ risk behaviors, and the relevance of the environmental clues such the use of coping strategies and social support in order to build up a good level of Self-Efficacy after the occurrence of an injury. All these data are relevant when designing both preventive and recovery interventions from the multidisciplinary as well as from the psychological point of view.

## Introduction

Sports injury is inherent to the sport. Different epidemiological studies showed the high prevalence of sport-related injuries (Azuara et al., 2014; Hurtubise et al., 2015; Padegimas et al., 2016). In addition, almost any athlete has suffered at least one injury during his/her life. Moreover, sport injury might not just only imperil his/her sports career but can also affect individual’s financial, social, and/or health issues (Udry and Andersen, 2008; Almeida et al., 2014).

Scientific literature highlights sport injury is a complex multidetermined phenomenon (Meeuwisse et al., 2007). One of the aspects that recent decades’ research has shown is the role of psychological factors in either triggering or preventing sports injuries (Maddison and Prapavessis, 2005; Almeida et al., 2014; Johnson et al., 2014). According to the highly influential Andersen and Williams’ Stress and Injury Model (Andersen and Williams, 1988; Williams and Andersen, 1998), stress is a key factor in the genesis of the injury. Stress response as a result of facing a demanding competitive situation produces an increase in the athlete’s muscle tension which, in turn, impairs motor coordination and reduces flexibility. In addition, the stress response can also narrow the visual span, provoking a loss of relevant peripheral information and increasing distraction. The model also assumes that the stress response is moderated by three main factors: athlete’s personality, history of stressors and coping resources. These psychosocial variables can either alleviate or aggravate the stress response and might eventually affect athlete’s vulnerability to injury (Petrie and Perna, 2004).

Likewise, according to Wiese-Bjornstal et al.’s (1998) “Integrated Model of Psychological response to Sport Injury,” when the athlete sustains an injury, it has also an effect on such psychological variables which, in turn, contribute to either promoting or hampering the rehabilitation process. Moreover, the interactive and cyclic nature of the relationships among these psychosocial variables may bring to a stress-injury-stress loop hard to interrupt, according to Olmedilla and García-Mas (2009) Global Psychological Model of Sports Injuries (see Olmedilla et al., 2015).

Among others, self-efficacy and anxiety are two determinants that can affect the stress response, and therefore sports injury. Bandura (1997) hypothesized that athletes with higher levels of self-efficacy set themselves more demanding goals, strive harder and maintain their commitment despite adversity. Therefore, these athletes may be more vulnerable to injury due to the assumption of greater challenges (Llewellyn and Sanchez, 2008; Llewellyn et al., 2008). To the contrary, self-efficacy has been usually considered to buffer psychophysiological reactivity. The higher confidence in one’s abilities is expected to reduce the negative impact of facing demanding situations (Bandura, 2012). Nevertheless, certain studies have found that higher levels of self-efficacy may increase psychological stress response, instead (Schönfeld et al., 2017). Moreover, sport self-efficacy cannot be considered separately from the athlete’s physical characteristics (Feltz and Lirgg, 2001), since the perception of one’s physical state is one of the four sources of one’s self-efficacy beliefs (Bandura, 1977).

Thus, it can be considered that self-efficacy does not have a unique and direct relationship with vulnerability to injury, depending, among other factors, on the type of strategies used to deal with demanding situations (Rubio et al., 2014), or of its relation with the trait anxiety of the athlete. In this sense, Johnson and Ivarsson (2011) analyzed the role of the different personality factors in the prediction of sports injuries using a sample of 108 male and female soccer players. Their results showed that high levels of trait anxiety along with low levels of self-efficacy were associated with increased risk of injury, as Bandura (1997) hypothesized.

One of the most relevant psychological variables present in the Andersen and Williams model (Andersen and Williams, 1988), is the anxiety personality trait, which determines the athlete’s trans-situational stress response (Williams and Andersen, 1998). Anxiety trait may cause the athlete to perceive different situations as stressors, increasing the physiological activation, and producing peripheral attentional narrowing (Rogers and Landers, 2005). Different pieces of research have generally shown that high levels of anxiety trait are related to the occurrence of injuries (Petrie, 1993; Lavallée and Flint, 1996; Williams and Andersen, 1998; Johnson and Ivarsson, 2011), although this relationship is not always consistent (Hanson et al., 1992; Maddison and Prapavessis, 2005; García-Mas et al., 2014). The complex set of bindings among trait anxiety and other psychosocial variables may help to explain these results (García-Mas et al., 2014).

Perceived social support is another Andersen and Williams’ model key variable within the coping resources that moderates the stress–injury relationship. The scientific literature shows inconclusive results in this regard. On the one hand, some studies indicate that the more extended the social support network of the athlete, the lesser is the risk of the injury (Hardy et al., 1991; Johnson et al., 2014; Petrie et al., 2014). However, other studies did not identify any relationship between these factors (Malinauskas, 2010; Rees et al., 2010). The inconsistency of these results is probably due to the fact that the mediating role of social support is more complex and fluctuating (Petrie et al., 2014), being mediated by its relations with anxiety (Covassin et al., 2014), or sports commitment (Scanlan et al., 2016).

Therefore, the present work aims to explore the relationships between personality factors, namely self-efficacy and trait anxiety, and coping resources, namely social support, which formed part of the Andersen and Williams model repeatedly highlighted by previous studies, and the physical characteristics of the athlete with regard to the onset of sports injuries. In order to address the complexity of the phenomenon, this paper uses a Bayesian Network analysis (BN; López-Puga et al., 2007) addressed to probabilistically explore the relationships between these variables. Currently, BN and Bayesian analysis are beginning to be used in sport psychology (Fuster-Parra et al., 2013, 2015, 2016; Gucciardi et al., 2016) aimed to construct graphical probabilistic models based on the underlying structure that connects the analyzed variables, in a complementary way to the use of traditional statistics.

Bayesian networks provides a statistical model describing the dependencies and conditional independencies from empirical data. Most studies have used cross-sectional designs assessing only statistical associations, rather than providing evidence of relationships of dependency and conditional independence between different variables. Moreover, BNs have the power of reasoning under an uncertain domain knowledge in a natural manner. They enable causal, intercausal, and evidential reasoning with domains concepts in a visually appealing fashion.

The process of a BN learning from data is a form of unsupervised learning, in the sense that the learner does not distinguish the dependent variable from the independent variables in the data, which is also an advantage when comparing to regression or structural equation modeling (SEM) models. Furthermore, BNs can be trained on the same structure with new data, showing an advantage with respect to SEM models which applied mainly to modeling linear relationships.

## Materials and Methods

### Participants

We surveyed a sample of 297 athletes who voluntarily participated in the study. They were from a wide range of different sports, including sports based on solo action, such as track and field, swimming or kayaking; co-operation sports such as rowing or artistic roller skating in pairs; opposition sports such as judo, fencing, boxing, or karate; and co-operation-opposition sports such as basketball, handball, or football. There were 239 men (80.50%) and 58 women (19.50%). The mean age was 25.10 (*SD* = 3.87; range = 21–38 years).

### Material and Instruments

The protocol to collect data on physical and injury-related variables was based upon the injuries’ definition and record system of Fuller et al. (2006) and Junge et al. (2009), which is used by the International Olympic Committee’s (IOC) and Fédération Internationale de Football Association’s (FIFA) competition surveillance studies.

The protocol consists of two sections. The first is related to the sport being practiced: modality, category, competitive level (competitiveN), training sessions per week (WeekF), hours per session, time practicing the sport, competitions per season, sport position (if applicable), and compensation (if applicable). The second concerned the incidence of injury: injured or not during the last 12 months (StateAthlete), number of injuries (quotaInjury), tissue affected, diagnosis, anatomical site, severity, athletic time lost, internal/external trigger event, treatment, season phase, and sustaining moment (training or competition). Details of the athletes’ gender (sex), weight, height, body scale, waist circumference, and body mass index (BMI) were also recorded.

Regarding the athletes’ competitive anxiety, we used the Spanish version (Spielberger et al., 2011) of the State-Trait Anxiety Inventory (STAI; Spielberger et al., 1970), which is composed of two subscales: Trait Anxiety (AnxietyR) and State Anxiety (AnxietyE). In this study, the Anxiety State scale reliability (Cronbach’s *α*) was 0.90 and the Anxiety Trait Scale was 0.87.

The Spanish version (Sanjuán et al., 2000) of the General Self-Efficacy Scale (Baessler and Schwarzer, 1996) was used in order to assess the athletes’ perceived self-efficacy (self-EfficacyT). This scale is composed of 10 five points Likert items and produced a Cronbach *α* value of 0.87.

Regarding the study of the athletes’ perceived Social Support, we used the Spanish version (Landeta and Calvete, 2002) of the Perceived Social Support Multidimensional Scale (Zimet et al., 1988). This scale includes family, friends, and other people social support (SupportS), and resulted in a global *α* value of 0.85.

The Spanish version (Kim et al., 2003) of the Approach to Coping Questionnaire (ACSQ; Kim and Duda, 1997) was used for assessing coping strategies. Locus of control (LOCUS) was assessed using the Spanish version (Pérez-García, 1984) of the Locus of Control Scale (Rotter, 1966). Risk-taking behavior was assessed using the Spanish version of Domain Specific Risk Taking Scale (DOSPERT; Weber et al., 2002). This scale includes two subscales: risk propensity (riskT) and risk perception (riskperceptionT).

### Procedure

Following IRB approval, the researchers contacted 30 different sports associations in the region of Madrid (Spain). Five of the 30 did not respond to our approach. The project was presented to those in charge of the sports associations that were interested in the research, in order to ask for their cooperation in recruiting participants. After they had agreed to communicate with different sports clubs and sports facilities, the researchers visited the premises and presented the project to the athletes who voluntarily decided if they wanted to participate. Those who did decide to participate signed the informed consent and were surveyed by the researchers. Each survey took approximately 20–30 min, and the data collection lasted 3 months. The collection of data for the whole population was carried out during the 3 months after the summer vacations.

### BN Modeling

In order to obtain the BN, we used the bnlearn package (Scutari, 2010) of the R language (R Development Core Team, 2016). To obtain the structure, there are two options either to select a single best model or to obtain some average model, which is known as model averaging (Claeskens and Hjort, 2008). Our model was learned by tabu algorithm (a search and score algorithm). The algorithm explores the search space starting from a network structure and adding, deleting, or reversing one arc at a time until the score can no longer be improved, a modified hill-climbing algorithm able to escape local optima by selecting a network that minimally decreases the score function. Neither expert knowledge nor prior knowledge of the system under study was taken into account in the model selection process in order to prevent the model from encoding the prior information instead of the information in the data.

The final model was obtained by repeating several times the structure learning. The results of several tabu searches, each starting from a different network was average; a large number of network structures was explored (1,000 BNs) to reduce the impact of locally optimal (but globally suboptimal) network learning. The score function implemented in bnlearn package is a score equivalence, i.e., networks that define the same probabilistic distribution are assigned the same score. The networks learned were averaged to obtain a more robust model. The averaged network structure was obtained using the arcs present in at least 85% of the networks, which gives a measure of the strength of each arc and establishes its significance given a threshold (85%).

Parameters were also obtained with the bnlearn package in the R language by performing a Bayesian parameter estimation using a Dirichlet distribution (Neapolitan, 2003). A conditional probability distribution was obtained for each node.

Bayesian networks are used to make inferences. Thus, it is necessary to know when influence flows from a node *X* to another *Y* via a node *Z*. Two variables *X* and *Y* in a BN are d-separated if, for every possible path between *X* and *Y*, there is an intermediate variable *Z* such that either: (1) the connection is serial (*X*→*Z*→*Y* or *X*←*Z*←*Y*) or diverging (*X*←*Z*→*Y*) and *Z* is instantiated, or (2) the connection is converging (*X*→*Z*←*Y*) and neither *Z* nor any of *Z*’s descendants have received evidence. To analyze the BN is necessary to know when the influence flows from one node *X* to another node *Y* via a node *Z*, in such a case it is said that the trail *X*→*Z*→*Y* is active. The global Markov property states that any node *X* is conditionally independent of any other node given its Markov blanket [which is composed by its parents, its children and the other children’parents (spouses)]. Any node in the BN would be d-separated from the nodes belonging to the non-Markov blanket given its Markov blanket.

### Validation

The BN was validated using a 10-fold cross-validation for BN, using a log-likelihood loss function, obtaining an expected loss of 9.187889. **Table 1** shows the area under the ROC curve (AUC), and the percentage correctly classified for the different features.

**Table 1:** AUCs and the percentage correctly classified for the different features by the Bayesian network (BN).

Variable name	Value	AUC	Accuracy
Sex	Men	0.8029	85.55
Sex	Women	0.8035	85.55
competitiveN	Registered	0.5460	89.63
competitiveN	Professional	0.6769	89.63
weekF	1–3	0.7868	85.55
weekF	4 or more	0.7886	85.55
quotaInjury	None	1	85.55
quotaInjury	1–3	0.7850	85.55
quotaInjury	4–6	0.6704	85.55
stateAthlete	WithInjury	1	100
stateAthlete	WithoutInjury	1	100
BMI	Underweight I	0.7268	69.63
BMI	NormalWeight	0.5744	69.63
BMI	Overweight	0.5602	69.63
BMI	ObesityI	0.6616	69.63
ACSQ	Low	0.6517	71.11
ACSQ	Moderate	0.6020	71.11
ACSQ	High	0.6229	71.11
LOCUS	Low	0.7154	51.11
LOCUS	Moderate	0.5487	51.11
LOCUS	High	0.6402	51.11
supportS	Low	0.8689	78.89
supportS	Moderate	0.7434	78.89
supportS	High	0.8087	78.89
selfEfficacyT	Low	0.9095	62.22
selfEfficacyT	Moderate	0.6971	62.22
selfEfficacyT	High	0.7718	62.22
riskT	Low	0.7012	65.93
riskT	Moderate	0.6770	65.93
riskT	High	0.9349	65.93
riskPerceptioT	Low	0.7195	62.22
riskPerceptioT	Moderate	0.6680	62.22
riskPerceptioT	High	0.7059	62.22
anxietyR	Low	0.7135	64.81
anxietyR	Moderate	0.6715	64.81
anxietyR	High	0.8539	64.81
anxietyE	Low	0.8334	72.96
anxietyE	Moderate	0.7958	72.96
anxietyE	High	0.9617	72.96

## Results

**FIGURE 1:**
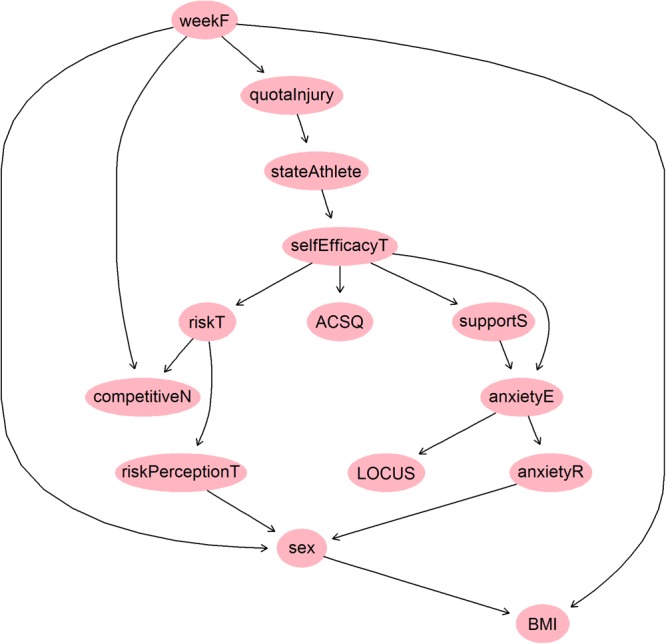
Directed acyclic graph obtained with bnlearn package on the studied variables.

In **Figure 1** we can see the directed acyclic graph (DAG) corresponding to the obtained BN. First, we have to outline the antecedent variable (also called “parent” node) of the graph, which is the frequency of sportive practice (weekF), which directly triggers four variables: the sex of the athletes, their competitive level (competitiveN), the frequency of sustained injuries (quotaInjury), and the BMI, which appears as the consequent variable, or “child,” node of this graph.

Also, we can observe the existence of two nodes, a secondary one corresponding to the state anxiety (which has as a “child” on the probability of occurrence of a certain level of anxiety trait), and another one that corresponds to Self Efficacy (SE). It can be observed that SE and Trait Anxiety (AR) are connected to two trails: (i) SE – Social Support – AnxietyE – AR, and (ii) SE – AnxietyE – AR, however, given AnxietyE then SE and AR would be d-separated. The StateAthlete (SA) and SE are directly connected. However, there is a direct trail SA – SE – ACSQ, and therefore when SE is instantiated SA and ACSQ would be d-separated.

Finally, we can see how the graph’s bottom nodes (without descendants) are (1) the use of coping strategies, and (2) the locus of control, as well as the intermediate situation of two more variables: (1) the risk tendency, related to the athlete’s competitive level, and (2) their perceived social support, connected with the anxiety and the locus of control.

**FIGURE 2:**
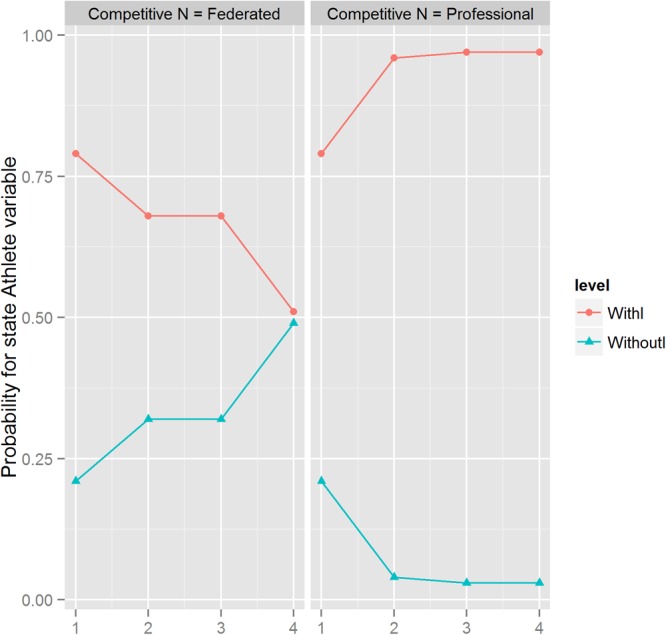
Step-by-step instantiations in the BN to maximize the state athlete feature values in high, low, and moderate states. The horizontal line represents the different steps from **Table 2**.

In **Table 2** it can be seen that by means of an instantiation the maximal hypothetical probability of suffering an injury is obtained, reaching 0.97 from the initial value shown by the studied population (0.79). In this case, we apply intercausal reasoning (when different causes of the same effect can interact) without taking into account the Markov blanket. Again, we choose from each step the variable and the state that induce the greatest increase in the likelihood of the state athlete variable in a high, low, and moderate state. **Figure 2** and **Table 2** summarize these results.

**Table 2:** Step-by-step instantiations leading to maximization of the likelihood of the state athlete variable (injured or not injured) in its high, low, and moderate state.

Step	Instantiate variable	Value	State Athlete = With Injury
1	None	–	0.79
2	selfEfficacy	Low	0.96
3	competitiveN	Professional	0.97
4	weekF	4 or more	0.97

			**State Athlete = Without Injury**

1	None	–	0.21
2	selfEfficacy	High	0.32
3	competitiveN	Registered	0.32
4	weekF	1–3	0.49

The instantiations modify the normal course of events of the BN, which is from the “top” to the “bottom” variables, by means of several “injections” of hypothetical data in some target variables in our study, these are the SE and the athlete’s state in terms of injury using a Markov blanket, in order to observe the changes made in the rest of the BN variables probability values.

This max value requires three successive steps. In the first and the most relevant one, the SE must be at the maximum probability in its low mode. The next two steps only increase the probability of occurrence of the injury by 0.001%. The first one is that the variable of competitive level goes to the maximum probability of to be a professional athlete, and the second, that the frequency of practice is at its maximum value possible (four times a week or more).

Second, also in **Table 2**, we may observe that when we instantiate the injured state variable to the minimum of probability, only a hypothetical maximum value of 0.49% can be reached (possibly mediated by the characteristics of the sample studied regarding their rate of “real” injuries). In order to obtain this hypothetical value, three successive steps were also required. Firstly, the variable AE needed to be placed at the maximum probability of its high state, while the competitive level must have been located in a registered member. This step implies a “jump” to more than 10% of probability. Finally, to reach the hypothetical maximum of 0.49%, it is necessary that the frequency of practice reaches a maximum of three times per week.

**FIGURE 3:**
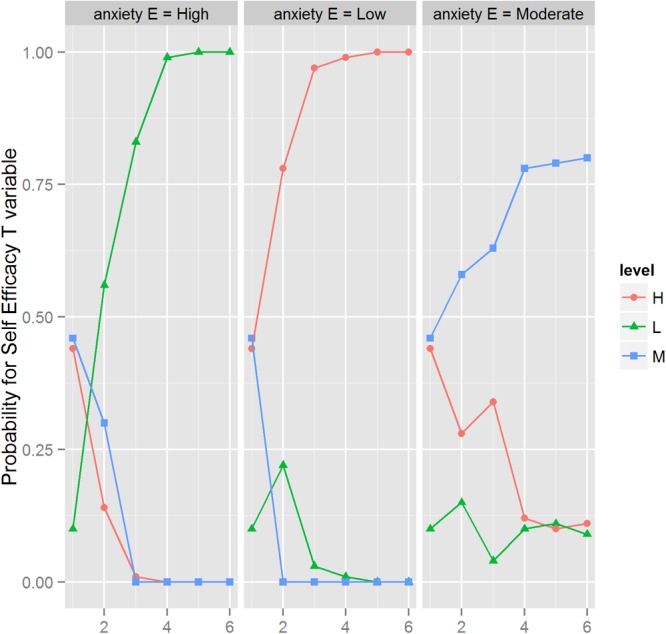
Step-by-step instantiations in the BN to maximize the self-EfficacyT feature values in high, low and moderate states. The horizontal line represents the different steps from **Table 3**.

In **Table 3** we can see the results of the second instantiation carried out on the SE variable. We choose from each step the variable from the Markov blanket [stateathlete (parent), supportS (child), anxietyE (child), ACSQ (child), and riskT (child)] and the state that induce the greatest increase in the likelihood of the SE variable in a high, low, and moderate state. **Figure 3** and **Table 3** show a summary of that.

**Table 3:** Step-by-step instantiations leading to maximization of the likelihood of the selfefficacy*T* variable in its high, low, and moderate state.

Step	Instantiate variable	Value	Self efficacy = High
1	None	–	0.44
2	riskT	High	0.78
3	stateAthlete	Without injury	0.97
4	supportS	High	0.99
5	anxietyE	Low	1.00
6	ACSQ	High	1.00

			**Self efficacy = Low**

1	None	–	0.10
2	supportS	Low	0.56
3	riskT	High	0.83
4	anxietyE	High	0.99
5	ACSQ	Low	1.00
6	stateAthlete	With injury	1.00

			**Self efficacy = Moderate**

1	None	–	0.46
2	anxietyE	Moderate	0.58
3	supportS	High	0.63
4	ACSQ	Low	0.78
5	stateAthlete	With injury	0.79
6	riskT	Moderate	0.80

This instantiation has tried to obtain the maximum probability of occurrence of the SE three states: high, medium, and low. When we instantiated the probability that SE would be in it higher value as much as possible, we needed five steps to reach 100%. The first step is the most relevant: the risk tendency had to be at its maximum, thus increasing the SE high value a 34%. The second, which added another 20% of probability, implies that the athlete’s state is not injured. The last three steps represent together just a 3% of an increase in the SE high probability. They are: (1) the athlete’s perception of social support had to be at the maximum in its High mode; (2) the athlete’s state anxiety had to be situated at its maximum of Low, and (3) that the use of coping strategies was situated at its maximum value of probability.

In the same **Table 3**, we can observe that to instantiate the SE at its 100% (maximum value) probability of being Low, we also needed to take five steps. The first, which increased the probability by some 50%, implied that the athlete’s social support is perceived at 100% in its Low mode. The second step, which increased the likelihood by another 27%, needed the Tendency to risk to be at its highest value. The third, which reached the 99% of probability, needed the anxiety state to be at its minimum value. The last two steps just increased a 0.001% to the final value of 100% and implied that the use of coping strategies was at its minimum value, while the state of the athlete should be injured.

Finally, as can also be seen in **Table 3**, in order to obtain the maximum probability that SE being at its hypothetical maximum of moderate values (80% from the initial value of 46%; it is not possible to reach the hypothetical value of 100%), we needed five steps. The first one implied that the state anxiety is at the maximum of moderate, obtaining an increase of 12%.

The next step implied that the perceived social support is at its High value, increasing the probability of SE (moderate mode) to the 63%. After this step, the use of the coping strategies had to be at Low, resulting in another 15% of an increase. The last two steps barely meant a 0.02% increase in the likelihood of the maximal value of the moderate label, and were the mode injured in the athlete’s state, and that the tendency to risk had to be at the 100% probability in its moderate mode.

## Discussion

When we analyze the results obtained through the use of BN, the first fact that strikes us is that the “parent” and “child” nodes do not have psychological characteristics: the frequency of practice (“top,” or antecedent), whose level directly triggers the probability of injury both in absolute and relative ways, and the BMI (found as a “bottom,” or descendant variable). Moreover, regarding the existence of nodes in the BN graph, self-efficacy appears to be the only relevant one. From this node (the SE), the graph divides itself into two “branches”: the first one consists of the behavioral risk and the competitive level, and the second is formed by the perceived social support and anxiety and attributional variables, leaving the use of coping strategies as another probabilistic outcome, but remaining isolated from the other variables. This is due to BNs are probabilistic models and cannot reliably be interpreted in a causal way if there are possible confounders or feedback loops that cannot be modeled; and that even in causal setting it is not always possible to identify the direction of all arcs from observational data. Hence, the existence of equivalence classes of BNs indistinguishable from a probabilistic point of view provides a simple proof that arc directions are not indicative of causal effects.

The role of SE in sports injuries has been extensively studied (De Pero et al., 2013; Rubio et al., 2014) but, in this study SE appears to have a quite central position regarding all types of variables, not only the psychological ones but also the somatic and epidemiological ones. In fact, SE appears as a consequence of the athlete sustaining an injury, as has been observed in other studies (Ardern et al., 2014; Everhart et al., 2015), and also as a probabilistic antecedent in four important aspects: (1) the anxiety and attributional components – repeatedly connected in some models as Andersen and Williams’s (1988) and Williams and Andersen (1998); (2) the coping strategies adopted when facing the injury, for dealing with both recovery and the return to play; (3) the degree of risk and competitive behaviors that can be assumed after the athlete’s appraisal – mediated by the SE – of his injury and its impact (a fact that only appears anecdotally in previous literature, such as in studies of Kontos et al. (2000) and Kontos (2004), and (4) although it seems obvious, it is confirmed that the frequency of practice determines the probability of occurrence of an injury (Hootman et al., 2007; Rechel et al., 2008), as well as the maximum probability is given at a registered member but not professional level of competition.

A global look at the set of variables studied and their location in the BN graph indicates a relevant data, regarding the global model of sports injuries (Olmedilla and García-Mas, 2009): probabilistic analysis has influenced the chain of events toward the psychological consequences of the injury. In other words, the psychological variables (self-efficacy, coping, locus of control, anxiety, and risk perception) studied have been placed in the graph as consequences of the injury, not as antecedents or triggers.

Although further empirical studies should confirm these results, it seems that an analysis based on the probability that certain events or variables were associated with a sports injury should focus on the psychological and social consequences of the injury, rather than its possible prevention, since the psychological variables do not appear as antecedents in the Bayesian graph. This data contrasts with other correlational studies analyzing similar data (Kontos, 2004; Short et al., 2004; Llewellyn et al., 2008). It means that from the probabilistic point of view, the difficulty of obtaining true data regarding the prevention of sports injuries might help to change the direction of the research toward the analysis of the consequences of sports injuries.

From an applied point of view, if we consider the sports injury to be almost an “inevitable” event in the athlete’s career, we can understand a possible probabilistic meaning of the BN graph: The chances of a good psychological outcome increase if we focus on the injury’s recovery, the return to sports practice and the prevention of possible later injuries. This approach would allow to correctly encompass the findings of this BN into the Olmedilla and García-Mas’s (2009) Global Psychological Model of Sport Injuries if we understood it not just sequentially, but as a way to express a feedback loop.

Although other studies indicated that extreme BMI values could be related to sports injuries (Tyler et al., 2006; Pollack et al., 2007), in the obtained BN and the instantiations made, BMI appears as an absolutely “bottom” variable without probabilistic connections with the rest of variables, but just as a consequence of the frequency of practice and the athlete’s sex.

When we study the instantiations made, the SE appears as central, in the same way as was depicted in the graph, as well as an antecedent and as a consequent at the same time, leading to think that the above mentioned idea of the “loop” may have some terrain to support. However, the second instantiation, which has been centered on the alteration of the SE values, appears a probabilistic link – which had not been highlighted in previous studies (Rubio et al., 2014) – with the tendency to risk, and connecting the level of SE with the fact that the athlete was not previously injured. Moreover, high level of social support and the use of coping strategies are also needed to maximize the probability of a high SE. This is confirmed when trying to obtain the minimum probability of the SE, which requires low social support, although the risk tendency remains at the same level – high – as when we “force” the SE value to the maximum.

Anxiety appears only with a low value, supporting the idea of its ambiguous role in sports injuries, as described in other studies (Lavallée and Flint, 1996; Ivarsson et al., 2013; Fernández-García et al., 2014; Johnson et al., 2014). At the same time, there is a probabilistic relationship between low SE and the injured state of the athlete.

On the whole, we may observe a landscape formed by several pictures: the nuclear role of the SE; the decreasing – at the conceptual level – role of the anxiety associated to the practice; the probabilistic independence of the risk behaviors, and the relevance of the use of coping strategies and social support in order to build up a good level of SE after the occurrence of an injury.

Our results should be taken into account when designing psychological intervention programs aimed to prevent sports injuries, beyond those addressed to the stress management (Ivarsson et al., 2015; Olmedilla et al., 2015, 2017). The lack of obtaining a network in which psychological variables appear as antecedents of sustaining the might mean that the better preventive approach should be pointing the focus on just the psychological complements to preventive programs from other professionals: doctors, physical therapists (Van Tiggelen et al., 2008; Johnson et al., 2014; Wiese-Bjornstal, 2014).

The limitations of this study are centered on the characteristics of the sample studied, which is composed of 80% athletes who sustained an injury during the last 3 years, and also gender biased toward males. Likewise, it should be made clear that the BN method is a probabilistic and retrospective study of events that have actually happened. Therefore, its predictive capacity rests fundamentally on the use of instantiations that can be carried out only when the BN has been correctly validated. And by using these instantiations forcing the probability of occurrence to 100%, for example, a very high contrast is being carried out with the probability observed in reality which appears in the BN. This limitation should not be overlooked when considering the analysis of the results obtained.

Regarding future developments in this line of studies, the focus should be the clarification of the relationship among the variables that have been shown to be sensitive, especially according to the instantiations made to modify the probability of occurrence of the injury and the levels of perceived self-efficacy. Thus, it would be plausible to design studies addressed to analyze the relationships between sport practice frequency and the competitive level, with the risk behaviors and competitive anxiety. Finally, it would be very interesting to study – using a qualitative–quantitative mixed methodology – the direct mechanisms which affect in building up of a certain value of SE, regarding the injury event in specific athletes, perhaps adding up observational-behavioral analysis.

## Ethics Statement

This study was carried out in accordance with the recommendations of Universidad Autonoma de Madrid IRB with written informed consent from all subjects. All subjects gave written informed consent in accordance with the Declaration of Helsinki. The protocol was approved by the Universidad Autonoma de Madrid IRB.

## Author Contributions

AO, VR, and AG-M made important contributions regarding the theoretical framework and interpretation of the data obtained. The first draft of the design was done by VR and checked out by AO and AG-M. The collection and tabulation of the data were carried out by CP. PF-P worked the implementation of the Bayesian Network, as well as the interpretation of the network, accompanied by AG-M. All authors contributed to the latest revisions of the manuscript, focusing in particular on the relevant aspects of the Discussion. Also, all authors are equally responsible for the reliability of the study and for the integrity of the research procedure carried out.

## Conflict of Interest Statement

The authors declare that the research was conducted in the absence of any commercial or financial relationships that could be construed as a potential conflict of interest.
